# Sterilizing Cocain Solutions

**Published:** 1900-06-15

**Authors:** 


### STERILIZING COCAIN SOLUTIONS.

Dr. Sidler, of Zurich, has made a lengthened and thorough investigation on the best method of sterilization of cocain solutions. Finding that anesthesia of the cornea ensued after the use of three drops of a 3 percent cocain solution (or one and a half grains to the ounce), and finding variations in the strength of the same, he concludes that cocain solutions lose their strength by sterilization; four or five drops of the solution used by Dr. Sidler after sterilization, and even more after repeated sterilization, were necessary to obtain the same effect in the same subject. This refers to sterilization by exposing the solution in an open bottle for twenty minutes to steam, whereas the process of rapidly heating the solution three times following to boiling point does not effect the efficacy of the solution. The immense importance of having absolutely sterile solutions is evident, but out of 470 solutions examined cocci or bacteria were found in twenty-four, proving beyond doubt that solutions, even when properly sterilized at first, can subsequently become re-infected. An examination of the antiseptic properties of different substances leads Dr. Sidler to exclude boric acid for want of efficacy and salicylic acid on account of its irritative properties, whereas a fresh sublimate solution of 1 to 10,000, and even an old carbolic solution of 1 to 100, were equal in their antiseptic properties. Dr. Sidler recommends alcoholic solutions as admirably adapted for the mother solutions, as they keep asceptic for years. He considers a solution of three to twenty grammes, or of five grains of cocain, to two-thirds of an ounce of a 40 percent solution of pure alcohol in water the best, and urges it being kept in a glass bottle previously sterilized, and recommends a new drop-bottle easy of sterilization for use whenever the solution is wanted. Thus every possible danger of infection can be avoided.—*Dental Record.*

